# Emulsion Quality and Functional Properties of Natural Emulsion Systems with Xanthan Gum as a Stabilizer and Carrier of Compounds Based on Enzymatically Modified Mutton Tallow and Hemp Oil

**DOI:** 10.3390/molecules31030431

**Published:** 2026-01-26

**Authors:** Małgorzata Kowalska, Magdalena Wozniak, Anna Zbikowska, Jerzy Szakiel, Paweł Turek

**Affiliations:** 1 Department of Chemistry, Casimir Pulaski Radom University, 26-600 Radom, Poland; wozniakmagdalena3@gmail.com; 2 Department of Food Technology and Assessment, University of Life Sciences SGGW (WULS-SGGW), Nowoursynowska 166, 02-787 Warsaw, Poland; anna_zbikowska@sggw.edu.pl; 3 Department of Non-Food Product Quality and Safety, Krakow University of Economics, 31-510 Kraków, Poland; szakielj@uek.krakow.pl (J.S.); turekp@uek.krakow.pl (P.T.)

**Keywords:** enzymatic interesterification, quality of emulsion, stability of emulsion, hemp oil, mutton tallow, xanthan gum, bioactive formulations, oxidative stress prevention

## Abstract

The aging population and increasing prevalence of oxidative stress-related diseases underscore the need for functional food and pharmaceutical formulations enriched with bioactive compounds. This study aimed to design sustainable emulsion systems incorporating enzymatically modified fats with enhanced functional and bioactive properties. Enzymatic interesterification was employed as an environmentally friendly alternative to chemical catalysis, enabling the transformation of natural lipids without generating undesirable trans isomers. The lipid phase was formulated from blends of hemp oil, a plant-derived source rich in polyunsaturated fatty acids with documented antioxidant potential, and mutton tallow, in an effort to valorize meat industry by-products. Systematic evaluation of emulsion stability, viscosity, and textural properties was conducted using Turbiscan analysis and texture profile analysis. The results demonstrated that xanthan gum concentration was the primary determinant of structural stability, physicochemical stability, and structural integrity of the emulsion systems. Formulation no. 38 (0.8% *w*/*w* xanthan gum) was identified as the statistically most stable system based on Turbiscan Stability Index values (TSI = 1.4). Although emulsions containing 1.0% *w*/*w* xanthan gum exhibited similarly low TSI values and slightly smaller final droplet diameters, formulation E38 showed the smallest increase in droplet size during storage (<1 µm), indicating superior resistance to structural changes over time. Fat composition showed minimal influence on emulsion behavior, suggesting that lipid selection should prioritize nutritional and bioactive value. These findings indicate that emulsions based on enzymatically modified fats and stabilized with natural polysaccharides can serve as physically stable systems with potential applicability in food, cosmeceutical, and pharmaceutical formulations intended for bioactive compound delivery.

## 1. Introduction

The global aging of populations has intensified the prevalence of oxidative stress-related diseases, including cardiovascular conditions, neurodegenerative disorders, and metabolic syndromes, underscoring the urgent need for functional food and pharmaceutical formulations enriched with bioactive compounds. Epidemiological evidence demonstrates that the dietary consumption of foods and ingredients rich in bioactive compounds with antioxidant activity provides substantial health benefits and significantly reduces the risk of stress-related and metabolic decline diseases. One of the key challenges in contemporary food technology, cosmetics, and pharmaceutical industries is to modify the physicochemical properties of lipids in a manner that enhances their versatility while preserving their bioactive potential and nutritional value. In this context, the process of interesterification has emerged as a particularly promising approach, enabling the conversion of natural fats, including lipid sources with documented antioxidant properties, into novel structures with altered functional and bioactive characteristics. Reformulation, defined as a chemical or enzymatic process involving the exchange of fatty acid residues between triacylglycerol molecules, facilitates the production of fats with a more desirable melting profile, improved plasticity, and, critically, enhanced oxidative stability, a key parameter for preserving bioactive compounds during processing and storage [1,2,3,4,5].

Traditionally, chemical interesterification employing alkaline catalysts (e.g., NaOH, KOH) has dominated industrial practice; however, such methods require elevated temperatures and generate difficult-to-remove by-products that compromise both product quality and environmental sustainability. In response to evolving industry requirements and intensifying regulatory pressure to implement sustainable solutions, enzymatic interesterification with lipases has gained considerable importance. This approach offers distinct advantages: greater selectivity, operation under mild conditions (40–60 °C, no organic solvents), and the utilization of immobilized enzymes enabling repeated use, thereby substantially reducing operational and environmental costs [6,7,8].

Therefore, it can be concluded that enzymatic interesterification exhibits a significant advantage over chemical methods primarily due to the possibility of conducting the process at substantially lower temperatures, which is crucial for the protection of unsaturated fatty acids. Reduced reaction temperatures limit the initiation of oxidative processes and the isomerization of double bonds, thereby preventing the degradation of polyunsaturated fatty acids, which are particularly sensitive to thermal stress. In contrast to chemical interesterification, which requires elevated temperatures and strong alkaline catalysts, the enzymatic process preserves the natural configuration of fatty acids as well as the integrity of accompanying bioactive compounds, such as tocopherols and phenolic compounds.

An additional advantage of enzymatic interesterification is the high specificity and selectivity of lipases, enabling controlled restructuring of triacylglycerol molecules without the formation of undesirable by-products. Chemical methods result in random fatty acid exchange, which may lead to a reduction in the nutritional value of the fat and necessitate intensive purification of the final product. Consequently, enzymatic interesterification allows for the production of structured lipids with enhanced oxidative stability and preserved bioactive potential, making it particularly suitable for applications in functional foods and health-promoting formulations. Moreover, enzymatic lipid modification eliminates the risk of trans isomer formation and significantly reduces energy and chemical inputs, aligning with the principles of green chemistry and sustainable development. Environmental and regulatory considerations underscore the imperative for sustainable lipid modification. In accordance with European Commission regulations and FDA guidelines [9], a comprehensive ban on partially hydrogenated oils (PHOs), the primary source of industrial trans isomers (EU Regulation 2019/649), has been in force for several years. Consequently, enzymatic interesterification represents one of the few viable alternatives for obtaining fats with appropriate physical structure without generating undesirable trans isomers. Modified fats, particularly interesterified variants, are extensively employed as structuring components in emulsion systems for both food and cosmetic products, where specific consistency, stability, and functionality of the lipid matrix are essential [10,11]. However, the modification of fat structure alone frequently proves insufficient to achieve the desired rheological properties and long-term stability. Consequently, formulations increasingly incorporate hydrophilic additives, such as thickening agents, which enable the further optimization of viscosity, structure, and physicochemical stability, while enhancing product functionality [12].

Polysaccharide texture modifiers play a pivotal role in stabilizing emulsions and conferring textural characteristics aligned with consumer expectations and regulatory standards [13,14,15]. Xanthan gum, a naturally occurring biopolymer widely utilized in industry, merits particular attention. This polysaccharide is especially valued for its exceptional rheological properties, which render it an effective stabilizer and texture modifier in water-based systems [16,17]. Its ability to maintain viscosity across a broad spectrum of temperatures and pH levels substantially enhances the texture and stability of diverse formulations [18]. Recent research has demonstrated that xanthan gum exerts beneficial effects on the viscosity and stability of fatty emulsions while acting synergistically with other ingredients [19,20,21]. Furthermore, the addition of this polysaccharide demonstrably reduces coalescence of the oil phase, thereby extending shelf-life and maintaining bioactive compound integrity throughout storage [22,23]. The combination of enzymatic lipid modification with innovative natural stabilizers, such as xanthan gum, represents a timely and promising strategy for designing emulsion systems with optimized physicochemical and functional properties that simultaneously address the need to eliminate partially hydrogenated fats and enable the implementation of environmentally responsible, bioactive-enriched solutions aligned with sustainable development objectives. The combination of mutton tallow and hemp oil represents a favorable base for interesterification due to their complementary fatty acid profiles. Mutton tallow provides oxidative stability and an appropriate lipid matrix structure, while hemp oil contributes polyunsaturated fatty acids and bioactive compounds with high health-promoting potential. Enzymatic interesterification enables the controlled redistribution of fatty acid residues within triacylglycerol molecules, thereby integrating the stability of the animal-derived fraction with the bioactive potential of the plant-derived fraction. A novel feature of this work is the use of a modified fat phase containing mutton tallow, which has made it possible to obtain a structural lipid base with a balanced composition, improved oxidative stability, and beneficial functional properties, conducive to the formation of stable and homogeneous emulsions capable of effectively delivering bioactive compounds.

This investigation examines the integrated influence of enzymatic interesterification and xanthan gum addition on the rheological properties, physicochemical stability, and bioactive preservation in model emulsion systems formulated from sustainable lipid sources. By correlating the properties of enzymatically modified fat mixtures with varying xanthan gum concentrations, this work aims to develop stable, functional emulsion matrices suitable for applications across food, cosmeceutical, and pharmaceutical industries.

## 2. Materials and Methods

The following principles were followed in the selection of methods and raw materials for testing: mutton tallow is a byproduct of meat production, representing a circular utilization of animal resources; enzymatic processes are mild, solvent-free, and energy-efficient, aligning with green chemistry principles; using natural polysaccharides (xanthan gum) reduces reliance on synthetic emulsifiers, promoting safer and biodegradable formulations. The conditions for preparing the raw materials and conducting the processes used in the work are specified below.

### 2.1. Materials

Mixtures of enzymatically modified fats were used in the study. The raw materials subjected to modification included mutton tallow (private supplier, Poland) and hemp oil (Oleofarm, Wrocław, Poland). The enzymatic interesterification process was carried out using lipase from *Rhizomucor miehei*, immobilized on Immobead 150 support, ≥300 U g^−1^ (Sigma-Aldrich, Saint Louis, MO, USA). The reaction was conducted at 60 °C for 6 h in a shaking incubator at a mixing speed of 200 rpm.

To increase the content of diacylglycerols and monoacylglycerols in the final product, a precalculated amount of water was added to the reaction, allowing for the desired level of polar fraction to be achieved (Table 1). The resulting mono- and diacylglycerols served as emulsifiers in the emulsions formulated with the modified fats. No synthetic emulsifiers were added to the system. The detailed interesterification procedure, which served as a preparatory stage for this study, has been described in detail in Ref. [24]. The interesterified fats obtained were prepared as mixtures with the following starting ratios of native fats of mutton tallow to hemp oil: mixture M1 (3:1); mixture M2 (3:2); mixture M3 (3:3); mixture M4 (2:3); mixture M5 (1:3). In this study, xanthan gum (XG), producer BASF (Ludwigshafen, Germany), was used as the emulsion material. The commercial preparation Euxyl K712 (Schülke & Mayr GmbH, Norderstedt, Germany), consisting of an aqueous solution of sodium benzoate and potassium sorbate, was used as a preservative in emulsions.

#### Emulsion Preparation Procedure

Each emulsion was prepared in the same way. Two phases were prepared: a fat phase and an aqueous phase. The fat phase contained the modified fat and, at the same time, an emulsifier (the sum of mono- and diacylglycerols, which was produced by enzymatic interesterification). The phase before homogenization was heated to a temperature of 50 °C. The aqueous phase contained water and a variable amount of texture modifier (xanthan gum) for the respective emulsion variant (Table 1). This phase, like the fat phase, was heated to 50 °C. The two phases were then mixed on a magnetic stirrer (approx. 30 s) and mechanically homogenized using an ULTRA-TURRAX T18 rotor-stator homogenizer equipped with an S18G-19G dispersing tool (IKA, Guangzhou, China). The homogenization process parameters were time: 4 min, speed of rotation: 18,500 rpm. The preservative was incorporated as the final component of the emulsion (0.1 mL). Each emulsion had a total weight of 100 g. Fifteen sustainable formulations were made (Table 1).

### 2.2. Methods

#### 2.2.1. Evaluation of Processes Occurring in Emulsions During Storage

The physical stability of the emulsion systems was assessed using a Turbiscan Lab Expert (Formulaction, L’Union, France), which operates based on Static Multiple Light Scattering (SMLS). This method enabled the detection of destabilization phenomena such as creaming, sedimentation, coalescence, and flocculation without the need for sample dilution. For analysis, approximately 20 mL of each freshly prepared emulsion was transferred into a cylindrical glass measurement cell and placed in the Turbiscan reading chamber. The instrument scanned the entire height of the sample (approximately 55 mm) at 40 µm intervals using a near-infrared light source (λ = 880 nm). Both transmitted (T) and backscattered (BS) light intensities were recorded as a function of sample height and time. The measurements were performed at 25 ± 1 °C for 30 days, with scans taken at regular time intervals. Destabilization processes were evaluated by analyzing the changes in BS and T profiles over time. The Turbiscan Stability Index (TSI) was calculated automatically by the software to provide a quantitative measure of sample instability, where higher TSI values indicated greater degrees of destabilization. According to [25], the index spans values from 0 (for highly stable systems) to 100 (for extremely unstable systems). Dispersions can be divided into five categories depending on the value of TSI. As suggested in [26], the following categories of TSI were used: A+—excellent system stability where the TSI value is <0.5; A—good system stability, where the TSI value is in the range 0.5–1; B—satisfactory system stability, where the TSI value is in the range 1–3; C—poor system stability, where the TSI value is in the range 3–10; D—unsatisfactory system stability, where the value of TSI is >10. According to the above categories, the value of the TSI coefficient was defined in the present study. In addition to the stability assessment, the Turbiscan values were used to estimate mean droplet size based on Mie theory, which relates the intensity of scattered light to the size and concentration of dispersed particles [27]. Since this approach did not require dilution, it preserved the native structure of the emulsion. Changes in backscattering profiles over time were interpreted to detect variations in droplet size due to coalescence or aggregation processes. A decrease in backscattering intensity typically indicates droplet growth (i.e., coalescence), while an increase may suggest flocculation or changes in particle concentration.

#### 2.2.2. Microscopic Assessment of Emulsions

The microstructure of the emulsion systems was examined after 24 h from manufacture and after storage (25 °C, 30 ± 1 days). For this measurement, a Genetic Pro optical microscope (Delta Optical, Warsaw, Poland) and a DLT Cam Pro camera (Delta Optical, Warsaw, Poland) were used (400× magnification).

#### 2.2.3. Texture Determination

Texture parameters of the emulsion systems (hardness and adhesive force) were determined using a CT3 Texture Analyzer (Brookfield Engineering Laboratories, Inc., Middleboro, MA, USA) and a 25.4 mm diameter nylon sphere-shaped probe. The investigation was carried out on emulsions placed in identical cylindrical dishes of 70 × 50 mm. The samples of each emulsion were penetrated once to a depth of 10 mm at a probe velocity (in both directions) of 2 mm/s. The texture determination was carried out at room temperature, 48 h after emulsion formation, and in triplicate; the results are presented as mean ± SD.

#### 2.2.4. Dynamic Viscosity Determination

The viscosity of the emulsions was measured using a Brookfield DV-III Ultra viscometer, model HA (Brookfield Engineering Laboratories, Middleboro, MA, USA), equipped with a Helipath stand. Investigations were conducted at 25 °C using a T-C spindle (No. 93) at variable speeds as follows: 2, 5, 10, and 15 rpm. Analyses were conducted in triplicate, 48 h after emulsion preparation, and the results are expressed as mean ± standard deviation (SD).

#### 2.2.5. Statistical Analysis of the Results

One-way ANOVA followed by Tukey’s HSD post hoc test (*p* < 0.05) was performed using Statistica 13 (StatSoft, Kraków, Poland). Tukey’s test compared TSI values between xanthan gum concentrations (0.6%, 0.8%, 1.0% *w*/*w*) within each fat blend (M1–M5). Different letters (a, b, c) denote statistically significant differences between means.

## 3. Results and Discussion

Xanthan gum is a polysaccharide produced by *Xanthomonas campestris* bacteria through aerobic fermentation [28,29]. It is one of the main texture modifiers used in the food industry [17]. It shows the ability to significantly increase the viscosity of aqueous solutions at relatively low concentrations [17]. Stabilization of O/W emulsion systems using xanthan gum occurs by increasing the viscosity of the aqueous phase and slowing down the creaming of the dispersed phase [30].

**Figure 1:**
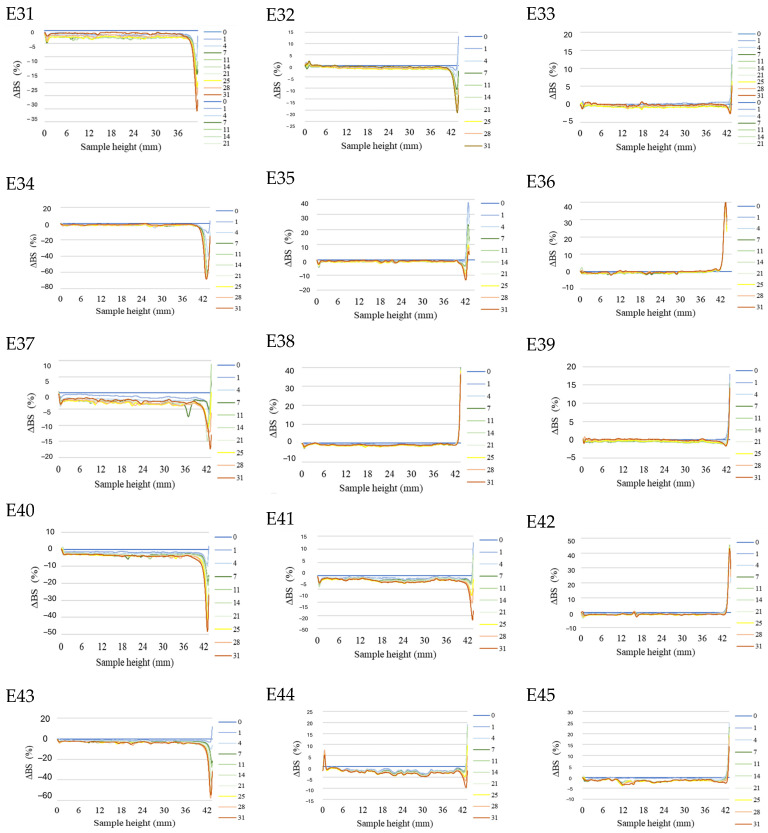
Profiles of changes in backscattered light intensity as a function of sample height for emulsions E31–E45 when stored for 31 days at 25 °C.

The profiles of the backscattered light intensity as a function of sample height in the reference mode for emulsions E31–E45, differing from each other by the type of fatty phase and the amount of xanthan gum introduced, are shown in Figure 1. In analyzing the changes in the backscattered light intensity of systems E31–E45, it was noted that the type of fatty phase had no effect on the emulsion stability; they showed no statistically significant differences from each other (*p* > 0.05).

However, it was observed that the changes recorded for the analyzed emulsion systems were significantly dependent on the applied concentration of the texture modifier. The most pronounced changes concerned systems E31, E34, E37, E40, and E43, containing the smallest analyzed amount of the studied texture modifier (0.6% *w*/*w*). For these emulsions, peaks were observed in the parts of the graphs corresponding to the upper part of the measuring vials. They showed that the concentration of dispersed phase droplets increased in this area, which indicates the creaming process taking place [31]. Similar observations were made for systems E32, E35, E41, and E44, containing 0.8% *w*/*w* xanthan gum. However, the peaks observed for these systems were clearly smaller (indicating less advancement of the creaming process) than for systems containing 0.6% *w*/*w* XG [32]. Systems containing 1.0% *w*/*w* XG (E33, E36, E39, E42, E45), as well as system E38, did not show any destabilizing changes. Regardless of the fatty phase used in the emulsion system, it was observed that, as the xanthan gum concentration in the system increased, a decrease in the mean ΔBS values in the middle parts of the graphs was recorded (E31 > E32 > E33, E34 > E35 > E36, etc.). The mean values of changes in backscattered light intensity in this area for systems E33, E36, E39, E42, and E45 containing 1.0% *w*/*w* XG did not exceed 2.0%, suggesting that flocculation and coalescence processes did not occur [27]. In contrast, for systems E31, E34, E37, E40, and E43, containing 0.6% *w*/*w* of this modifier, the mean ΔBS values in the middle part exceeded 3.0%. Thus, it can be concluded that increasing the concentration of the texture modifier in the studied range decreased the kinetics of coalescence and flocculation processes.

**Figure 2:**
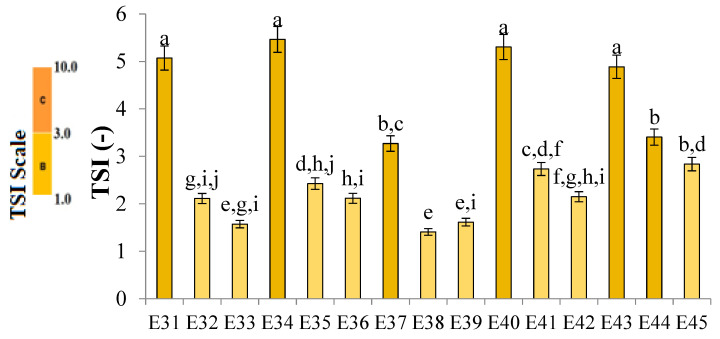
TSI values for systems E31–E45, stored for 31 days at 25 °C. a–j—different letters denote statistically significant differences between the means (*p* ≤ 0.05).

These results were confirmed by the stability factor results (Figure 2). TSI values of systems containing 0.6% *w*/*w* XG were in the range 3.3–5.5. Only the value obtained for system E37, containing modified fat in equal proportions of animal fat and vegetable oil, was statistically significantly lower (*p* ≤ 0.05) than the values obtained for the other systems (E31, E34, E40, and E43). It was observed that for each modified fat blend used as a fatty phase of the emulsion, an increase in xanthan gum concentration to 0.8% *w*/*w* resulted in a statistically significant (*p* ≤ 0.05) decrease in TSI values and thus an increase in system stability. Further increases in texture modifier concentration (up to 1.0% *w*/*w*) did not cause a statistically significant (*p* > 0.05) change in the stability coefficient of the systems. Among the analyzed systems, emulsion E38 exhibited the lowest TSI value (TSI = 1.4). However, it should be noted that emulsions containing 1.0% *w*/*w* xanthan gum (E33, E39, E42, and E45) showed similarly low TSI values (≈1.7–1.9) and showed no statistically significant differences from E38 (*p* > 0.05). Therefore, E38 can be described as, statistically, the most stable formulation, although several 1.0% XG systems demonstrated comparable stability. The values obtained for the E39 and E33 systems were higher, but not statistically significantly different (*p* > 0.05) from the value obtained for the E38. On the other hand, all systems containing the lowest analyzed concentration of XG (E31, E34, E37, E40, and E43) were classified under category C, indicating their poor stability. E44 was also classified under this category. The remaining emulsions (E32, E33, E35, E36, E38, E39, E41, E42, and E45) were classified under category B (satisfactory stability).

As indicated in [33], when the xanthan gum concentration used in emulsion systems is above 0.3% *w*/*w*, these systems can remain stable for several days or even months. The results obtained in the study of the stability of E31–E45 systems produced on the basis of modified fats are in agreement with the results obtained in the presented work.

**Figure 3:**
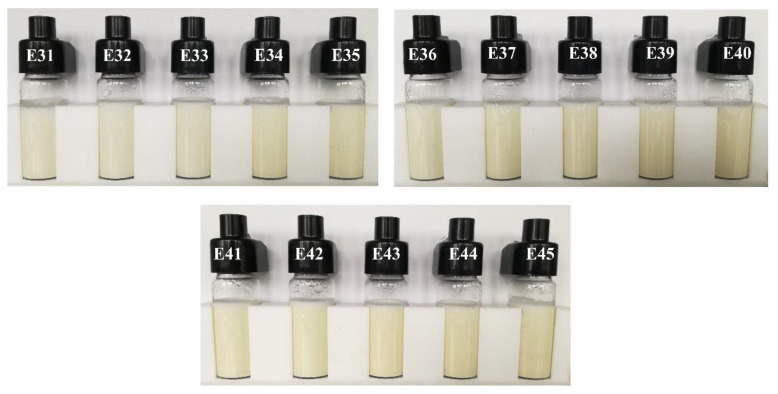
Photographs of systems E31–E45 after 30 days of storage at 25 °C.

Photographs of systems E31–E45 after 31 days of storage are shown in Figure 3. Visual evaluation of these emulsions clearly confirmed the high durability of the systems. No changes were observed in the visual evaluation of the presented emulsion systems.

**Figure 4:**
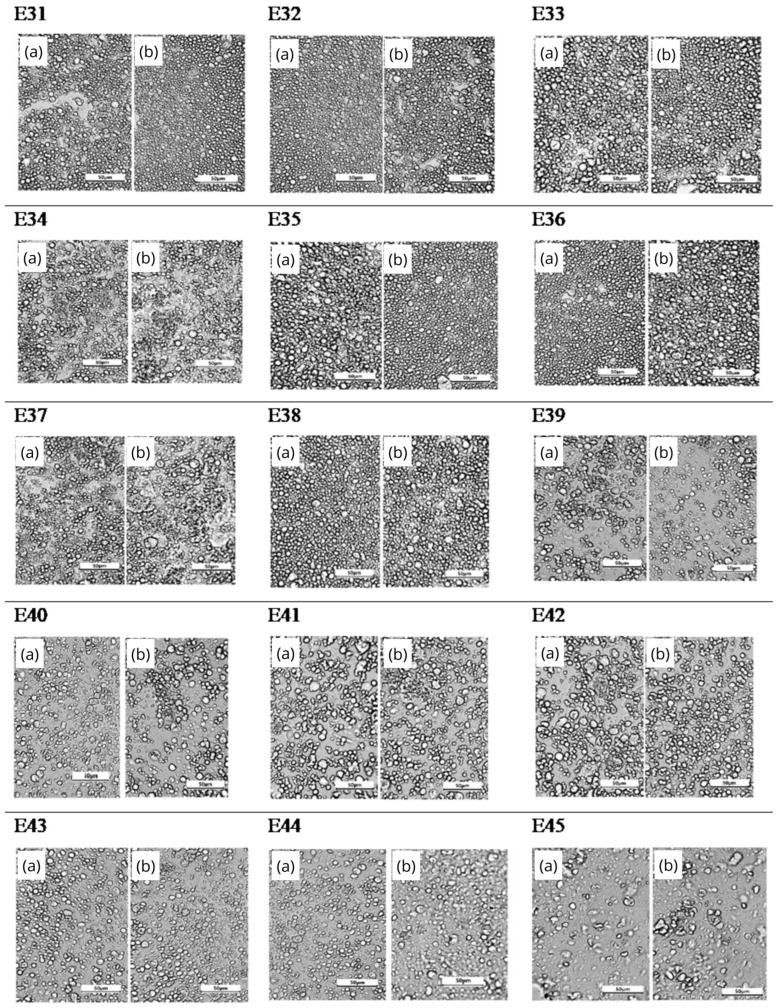
Microstructure of E31–E45 systems: (**a**) 24 h after manufacture and (**b**) after 30 days of storage at 25 °C (400×). Scale bar: 50 μm.

Figure 4 shows photographs of the microstructure of emulsion systems E31–E45 taken 24 h after manufacture and after 30 days of storage. Analyzing the microstructure changes in the XG-stabilized systems, it was found that all systems were characterized by a relatively low degree of polydispersity after manufacture. The droplets of the dispersed phase were uniformly dispersed in the continuous phase and were characterized by a relatively small diameter and high symmetry. After the storage period, no clear changes in the microstructure image were registered for all analyzed systems. No dependence of the microstructure image on the viscosity modifier concentration, as well as on the type of fatty phase, was observed.

**Figure 5:**
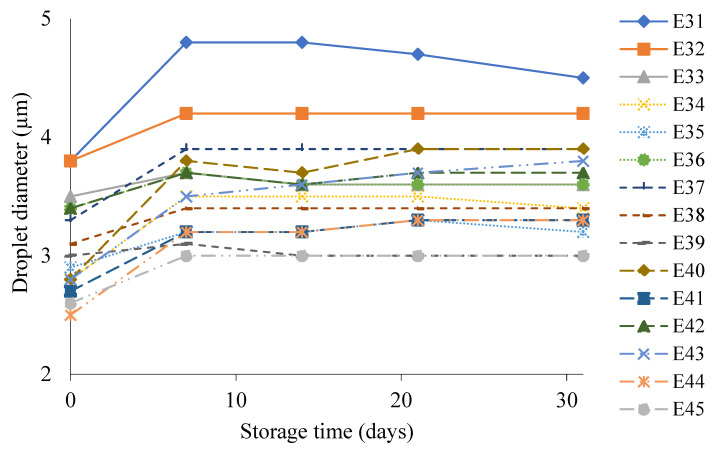
Droplet diameter of dispersed phase of E31–E45 systems when stored for 30 days at 25 °C.

The diameters of the dispersed phase droplets of systems E31–E45 during storage are shown in Figure 5. All systems stabilized by xanthan gum after manufacture were characterized by droplets with relatively small diameters, ranging from 2.5 to 3.8 µm. For all systems, a slight increase in droplet diameter occurred after the storage period. Among the analyzed systems, the highest increase in the droplet diameter in the range of 0.6 to 1.1 µm, which occurred after 31 days of emulsion storage, was characteristic of the systems containing the lowest concentration of XG (0.6% *w*/*w*). As the XG concentration increased (0.8 and 1.0% *w*/*w*), slightly lower increments in the diameter of the dispersed phase droplets were recorded: the range for the 0.8% *w*/*w* XG concentration being from 0.3 for E35 to 0.8 µm for E44, and for the 1.0% *w*/*w* XG concentration, from 0.0 for E39 to 0.4 µm for E45, respectively. After storage, emulsions E39 and E45 (1.0% *w*/*w* XG) exhibited the smallest final droplet diameter (approximately 3.0 µm). In contrast, emulsion E38 (0.8% *w*/*w* XG) showed a slightly larger final droplet size, but was characterized by the smallest increase in droplet diameter during storage (<1 µm), indicating superior resistance to droplet growth. Ref. [34] obtained opposite results of droplet size determination in the analyzed XG-stabilized O/W mayonnaise emulsions. These emulsions were characterized by an XG concentration of 0.5% *w*/*w*, while the authors used egg yolk as the emulsifier and the fatty phase was a fat blend of soybean oil and palm kernel olein in a mass ratio of 70:30. After a 30-day storage period of the systems at 25 °C, a significant increase in the droplet size of the dispersed phase from 11 µm to 20 µm was recorded.

**Figure 6:**
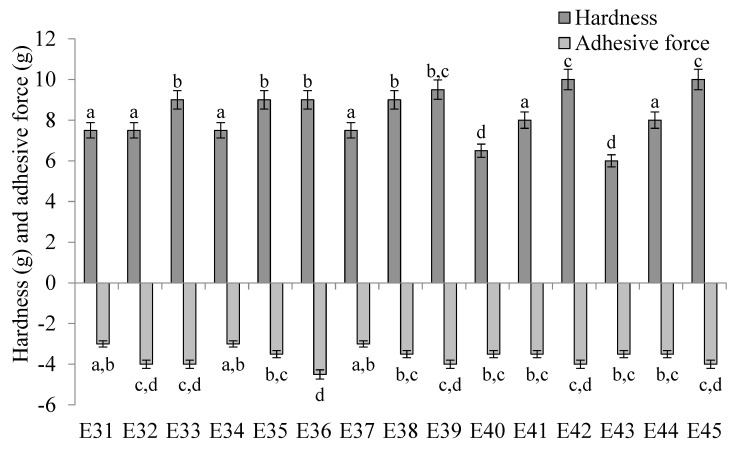
Hardness and adhesive force of E31–E45 systems determined 48 h after manufacture. a–d—different letters denote statistically significant differences between the means for individual texture parameters (*p* ≤ 0.05).

Figure 6 shows the values of the hardness parameter and the adhesive force of the E31–E45 emulsion systems. The hardness of the produced systems was similar and ranged from 6.0 to 10.0 g. It was observed that the applied XG concentration in the emulsion systems affected the hardness of these systems. The hardness values characterizing emulsions containing minimum and maximum XG content, i.e., 0.6 and 1.0% *w*/*w*, were statistically significantly (*p* ≤ 0.05) different for each of the fatty phases used. However, for differences in texture modifier concentration, i.e., 0.6 and 0.8% *w*/*w* or 0.8 and 1.0% *w*/*w*, the hardness values obtained for systems E31–E39 were not statistically significantly (*p* > 0.05) different from each other. Statistically significant (*p* ≤ 0.05) differences were recorded for emulsions E40–E45 containing fatty phases in which the hemp seed oil content was the highest. When analyzing the effect of the type of fatty phase used in the emulsion, no close correlation was observed between this parameter and the hardness or adhesive force values obtained.

In analyzing the adhesive force determined for the studied systems, it was found that the values obtained for all systems slightly differed from each other and ranged from −3.0 to −4.5 g. It was observed that the values of adhesive force obtained for emulsions containing the same concentration of XG, but differing in the type of fatty phase, showed no statistically significant differences from each other (*p* > 0.05). The adhesive force determined for the emulsions corresponded slightly with the concentration of xanthan gum used in these systems. In general, a slight increase in adhesive force was observed with increasing XG concentration. However, for systems with the same fatty phase but different XG content, the determined values of adhesive force showed no statistically significant differences (*p* > 0.05).

**Figure 7:**
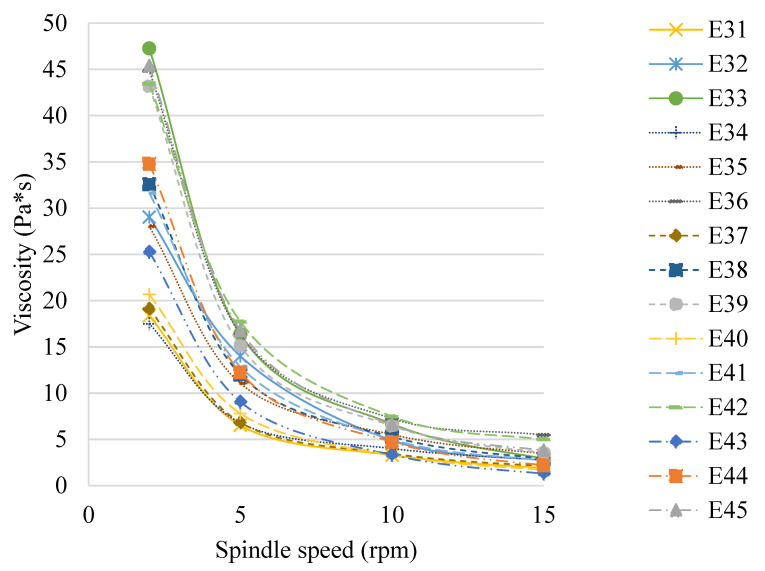
Viscosity of E31–E45 systems determined 48 h after preparation.

The viscosity values of the E31–E45 systems determined 48 h after their preparation are shown in Figure 7. A significant effect of the viscosity modifier (XG) concentration used on the viscosity of the emulsion systems was observed. The viscosity of the produced systems, regardless of the fatty phase used and the spindle speed, increased with increasing XG concentration (E31 < E32 < E33, E34 < E35 < E36, etc.). The type of fatty phase used had little effect on the viscosity of the analyzed emulsions. The viscosity of the studied systems determined at a rotation speed of 2 rpm was in the range of 18–25 Pa*s for a texture modifier concentration of 0.6% *w*/*w*, 29–35 Pa*s for 0.8% *w*/*w*, and 43–47 Pa*s for 1.0% *w*/*w*. The systems E31–E45 stabilized with xanthan gum were characterized as non-Newtonian shear-thinning (pseudoplastic) liquids [30]. For the systems analyzed, an increase in the degree of pseudoplasticity was observed with increasing xanthan gum concentration. Similar results were obtained by [35], who investigated the effect of xanthan gum on, among others, the rheological properties of corn oil-based emulsion systems.

On the basis of the results obtained, it was found that all the systems containing xanthan gum exhibited relatively good stability; however, the stability of emulsion systems stabilized by xanthan gum was clearly dependent on the concentration of texture modifier used. Among all analyzed systems, emulsion E38 exhibited the most favorable overall stability profile, combining the lowest TSI value with minimal droplet size increase during storage, despite not having the smallest final droplet diameter. This product, among others, was characterized by a small increase in the diameter of the dispersed phase droplets <1 µm and an ordered arrangement of the dispersed phase droplets. In our opinion, such a small increase during storage clearly gives the green light to expand research into the functionality of model emulsion systems, such as pharmaceutical preparations, cosmetic products, and functional food products.

The results obtained in this study confirm the validity and effectiveness of the applied texture modifier, as well as modified fats, in the formation of stable emulsion systems. This constitutes further evidence consistent with the findings of other authors who used blended fats as the lipid base and compared them with interesterified fats, obtaining clear conclusions consistent with our results, indicating the superior stability of emulsion systems based on interesterified fats [36].

## 4. Conclusions and Summary

The stability of emulsion systems stabilized with xanthan gum demonstrated a clear dependence on the concentration of the texture modifier used. Formulation no. 38 (0.8% *w*/*w* xanthan gum) was identified as the statistically most stable formulation based on Turbiscan Stability Index values, while formulations containing 1.0% *w*/*w* xanthan gum exhibited similarly low TSI values but smaller final droplet diameters. Importantly, E38 showed the smallest increase in droplet size during storage, indicating superior resistance to structural changes over time. This optimal formulation was characterized by minimal increase in droplet diameter of the dispersed phase (less than 1 µm) and uniform arrangement of droplets within the continuous phase. Those parameters are critical for maintaining long-term stability and functional properties of bioactive-enriched formulations.

The choice of lipid mixture composition showed minimal impact on emulsion behavior; therefore, fat selection in designing such systems should be primarily guided by nutritional and bioactive value rather than physicochemical considerations. Hemp oil, employed in this study as the plant-derived lipid component, is recognized as a source of polyunsaturated fatty acids with documented antioxidant and anti-inflammatory properties. Enzymatically modified mutton tallow, as a means to valorize animal by-products through enzymatic interesterification, provides a sustainable lipid matrix that preserves oxidative stability crucial for bioactive compound preservation during processing and storage. The combination of enzymatically modified fats with enhanced oxidative stability and xanthan gum as a natural stabilizer enables the formulation of stable, structured, and sustainable emulsion matrices with the potential to deliver bioactive compounds effectively to consumers.

This methodology represents an innovative approach to addressing oxidative stress-related diseases and metabolic decline through functional food and pharmaceutical formulations. By leveraging enzymatic processing under mild conditions, the approach avoids chemical catalysis that could compromise bioactive compounds, thereby maximizing their retention and bioavailability. The integration of animal by-products and plant-derived lipids enriched in bioactive compounds, combined with natural polysaccharide stabilizers, demonstrates the feasibility of developing functional emulsions capable of delivering antioxidant and health-promoting benefits. The overall evaluation of all tested emulsions indicates relatively good stability after 30 days of storage, with 73% of formulations achieving satisfactory or better stability ratings (TSI categories A+ to B). The necessity of monitoring the stability of such systems arises from their dynamic nature, where even subtle physicochemical changes may affect bioactive compound integrity and functional efficacy.

The findings confirm that emulsified systems incorporating enzymatically modified fats with preserved oxidative stability and natural stabilizers can be effectively developed for sustainable and health-promoting applications. This approach aligns with sustainable development goals by promoting resource efficiency, reduced chemical input, environmentally responsible production, and the valorization of agricultural and animal industry residues.

In summary, the conclusions drawn from this study provide a solid foundation for the practical implementation of the developed emulsion systems. In particular, the demonstrated stability of interesterified fat-based emulsions supports their potential application in functional food products as well as in topical formulations requiring controlled texture and long-term physicochemical stability. These findings indicate that such systems may serve as promising lipid carriers for bioactive compounds. Future research should focus on in vitro digestion models and bioavailability studies to evaluate lipid structuring effects on nutrient release, absorption, and metabolic behavior, thereby facilitating the translation of the proposed formulations into industrial and nutritional applications.

## Figures and Tables

**Table 1:** Composition of emulsions produced.

Emulsion Symbol	Type of Fatty Phase	Texture Modifier Content [% *w*/*w*]	Emulsifier Content [% *w*/*w*] *
E31	M1	A (0.6)	7.2 ± 1
E32		B (0.8)	
E33		C (1.0)	
E34	M2	A (0.6)	
E35		B (0.8)	
E36		C (1.0)	
E37	M3	A (0.6)	
E38		B (0.8)	
E39		C (1.0)	
E40	M4	A (0.6)	
E41		B (0.8)	
E42		C (1.0)	
E43	M5	A (0.6)	
E44		B (0.8)	
E45		C (1.0)	

* the value that results from the calculation of the non-polar and polar phase content of the enzymatically modified lipid mixtures produced, calculated as the sum of the mono- and diacylglycerols which formed the emulsifier in the emulsions produced (interesterification procedure) [24].

## Data Availability

The datasets generated during the current study are publicly available in the [Repozytorium Otwartych Danych Badawczych Uczelni Krakowskich] repository at https://uek.rodbuk.pl/dataset.xhtml?persistentId=doi:10.58116/UEK/UGITFJ (accessed on 16 December 2025).
